# Data field theory: a geometric framework for learning on Riemannian manifolds with synthetic validation and limitation analysis

**DOI:** 10.3389/fdata.2026.1752468

**Published:** 2026-06-11

**Authors:** Mohammadreza Nehzati

**Affiliations:** DFT Labs, Knoxville, TN, United States

**Keywords:** critical phenomena, data field theory, geometric machine learning, hyperbolic geometry, manifold learning, renormalization group

## Abstract

**Introduction:**

Conventional machine learning treats learning as parameter optimization, lacking a first-principles framework for phenomena like criticality, generalization, and causal structure. We introduce Data Field Theory (DFT), a mathematical framework modelling learning as the evolution of a data field governed by stochastic partial differential equations on Riemannian manifolds. This work aims to validate DFT's core predictions in settings where its geometric assumptions hold, while honestly assessing its empirical limitations.

**Methods:**

We formulate learning as a field $\varphi :M\times {\mathbb{R}}_{\geq 0}\to {\mathbb{R}}^{k}$ evolving on a spherical manifold. To test DFT, we implement a hierarchical classification task using synthetic data drawn from von Mises-Fisher distributions, ensuring match with the manifold geometry. We derive four key predictions: (1) critical exponents near concept formation, (2) a spectral robustness law linking Eigen gaps to out-of-distribution (OOD) error, (3) finite-speed causal propagation from hyperbolic regularization, and (4) approximate rotational equivariance via a Ward identity. We also conduct a preliminary real-data experiment projecting MNIST digits onto the sphere.

**Results:**

Synthetic experiments validate all four predictions: (1) Correlation length diverges as $\xi \left(t\right)\sim |t-t_{c}{|}^{-\nu }$ with ν = 0.63 ± 0.04, accompanied by 1/f fluctuations; (2) OOD generalization error scales as $\epsilon_{\text{OOD}}\propto m_{\text{gap}}^{-2}$ (ρ = −0.78, *p* < 10^−6^); (3) Causal propagation speed *c*_eff_ = 0.98 ± 0.03 (theory maximum *c*_max_ = 1.0) under hyperbolic regularization; and (4) Ward identity residual *R* = 0.0032 ± 0.0008 converging as *R*∝*h*^1.02^. However, on real-world MNIST-sphere data, DFT achieves only 15.7% accuracy versus 51.7% for k-NN, revealing critical limitations.

**Discussion:**

DFT successfully predicts emergent phenomena criticality, spectral robustness, bounded causality, and approximate equivariance under ideal geometric conditions, supporting its theoretical validity. The poor real-data performance highlights key gaps: the current framework lacks adaptive metric learning, noise robustness, and hierarchical feature extraction present in real images. These results establish DFT as a principled mathematical foundation for learning as field dynamics while clearly delineating necessary extensions for practical applicability.

## 1 Introduction

Modern artificial intelligence has achieved remarkable empirical success through deep learning architectures with billions of parameters. However, this success has largely been heuristic, characterized by extensive trial-and-error rather than first principles. The dominant paradigm formulates learning as high-dimensional parameter optimization: $\theta^{*}=\arg\min_{\theta \in {\mathbb{R}}^{D}}{𝔼}_{\left(x,y\right)\sim D}\left[L\left(f_{\theta }\left(x\right),y\right)\right]$, which, while computationally powerful, lacks the theoretical coherence and predictive laws characteristic of mature scientific theories.

### 1.1 Related work and novel contributions

Our work builds upon several research traditions while making distinct contributions. Geometric deep learning explores neural architectures that respect underlying data symmetries (Bronstein et al., 2021). Neural field theories model cortical dynamics as continuous fields (Coombes et al., 2014), while the critical brain hypothesis suggests neural systems operate near phase transitions (Chialvo, 2010; Beggs and Plenz, 2003). Hyperbolic embeddings leverage negative curvature for hierarchical representations (Nickel and Kiela, 2017). Our contribution synthesizes these approaches within a unified field-theoretic framework with explicit mathematical foundations and reproducible synthetic validation. Unlike previous work, DFT provides a complete mathematical derivation from variational principles, explicit testable predictions derived from first principles, a reproducible experimental framework with a concrete learning task, and validation against multiple alternative dynamical systems to isolate DFT-specific effects.

### 1.2 Paper structure

Section 2 presents the mathematical foundations of DFT. Section 3 details the experimental framework, including the learning task, computational implementation, and statistical methodology. Section 4 presents experimental validation of the four predictions on synthetic data. Section 5 discusses implications, limitations (including the real-data limitation analysis), and future directions. Supplementary derivations and implementation details are provided in the Appendices A1–A4.

## 2 Mathematical foundations of data field theory

### 2.1 Data field formulation

** Definition 2.1 (Data Field)**. Given a compact Riemannian manifold (M, *g*) of dimension *d*, a *data field* is a time-dependent vector-valued function: $\phi :M\times {\mathbb{R}}_{\geq 0}\to {\mathbb{R}}^{k}$, where *k* represents the dimensionality of the representation space. The field ϕ(*p, t*) encodes the state of the learning system at position *p* ∈ M and time *t*.

The dynamics of the data field follow from variational principles. We define the Ginzburg-Landau free energy functional as Equation 1:


$$\begin{array}{l} F\left[\phi \right]=\int_{M}\left[\frac{1}{2}∥\nabla \phi {∥}_{g}^{2}+V\left(\phi \right)\right]d\text{vo}{\text{l}}_{g}, \end{array}$$ (1)

where the potential *V*(ϕ) is $V\left(\phi \right)=-\frac{\alpha }{2}∥\phi {∥}^{2}+\frac{\beta }{4}∥\phi {∥}^{4}$ with α,β > 0. This is the standard ϕ^4^ theory that exhibits spontaneous symmetry breaking.

** Assumption 2.2 (Learning as Energy Minimization)**. *Intelligent behavior corresponds to the minimization of* *F*[ϕ] *under constraints imposed by data* *J*(*p, t*) *and noise* ξ(*p, t*).

The field evolution follows gradient descent with stochastic fluctuations as Equation 2:


$$\begin{array}{l} \frac{\partial \phi }{\partial t}=-\Gamma \frac{\delta F\left[\phi \right]}{\delta \phi }+J\left(p,t\right)+\sigma \xi \left(p,t\right), \end{array}$$ (2)

where Γ > 0 is a mobility coefficient, *J*(*p, t*) represents external input signals (data), ξ(*p, t*) is space-time white noise, and σ controls noise amplitude.

### 2.2 Hyperbolic regularization for causal structure

To ensure finite propagation speed and numerical stability, we introduce hyperbolic regularization via the telegraph equation:


$$\begin{array}{l} \tau \partial_{tt}\phi +\partial_{t}\phi =-\Gamma \frac{\delta F\left[\phi \right]}{\delta \phi }+J+\sigma \xi , \end{array}$$ (3)

where τ > 0 controls inertia.

** Proposition 2.3** (Finite Propagation Speed). *Solutions of Equation 3* *satisfy finite propagation speed* $c\text{max}=\sqrt{\Gamma /\tau }$: *if initial data* ϕ(*p*, 0) *and* ∂_*t*_ϕ(*p*, 0) *have compact support within* *K* ⊂ M, *then for* *t* > 0, ϕ(*p, t*) *has support within the* *c*max*t-neighborhood of* *K*.

### 2.3 Spectral analysis and stability

The Hessian of the free energy plays a crucial role in stability analysis: $H\left[\phi \right]=\frac{\delta^{2}F\left[\phi \right]}{\delta \phi^{2}}=-\Delta g+V^{\prime \prime }\left(\phi \right)$.

** Definition 2.4** (Spectral Gap). For an equilibrium field configuration ϕ^*^, the spectral gap is $m_{\text{gap}}^{2}=\lambda_{2}\left(H\left[\phi^{*}\right]\right)$, *where λ_2_ denotes the smallest positive eigenvalue (excluding zero modes from continuous symmetries). The spectral gap depends on the field configuration and therefore varies with the potential parameter α (Binder, 1981)*.

** Theorem 2.5** (Generalization Bound). *For a data field ϕ with spectral gap m_gap_, the out-of-distribution generalization error is bounded by:* $\epsilon_{\text{OOD}}\leq \frac{CL^{2}\Delta_{\text{shift}}}{m_{\text{gap}}^{2}}+C^{\prime }e^{-m_{gap}D}$, *where L is the Lipschitz constant of the decision function, Δ_shift_ measures distribution shift,* *D is distance to domain boundary, and* *C, C*′ > 0 *are constants depending only on manifold geometry*.

## 3 Experimental framework and methods

### 3.1 Learning task specification

To ground DFT in a concrete learning problem, we define a hierarchical classification task on the manifold M = *S*^2^. Consider a taxonomy with *C* = 8 categories organized in a binary tree of depth *L* = 3. Each category *c* corresponds to a region $C_{c}\subset S^{2}$ centered at prototype $\mu_{c}\in S^{2}$.

** Definition 3.1** (Data generation). *Data points* (*x, y*) *are generated as: (1) Sample category* *c*~*Uniform{1,* …, *C*}; *(2) Sample x*~vMF(μ_*c*_, κ), *the von Mises-Fisher distribution with mean* μ_*c*_ *and concentration κ; (3) Set label* *y* = *c*. *The von Mises-Fisher distribution on* *S*^2^ *has density* *p*(*x*|μ, κ) ∝ exp(κμ^⊤^*x*).

### 3.2 Operational definition of concept formation

To avoid circularity in critical phenomena analysis, we define concept formation time *tc* independently of correlation statistics:

** Definition 3.2** (Concept formation time). *For a field trajectory* ϕ(*t*), *define* $tc=\inf\left\{t>0:I\left(\phi \left(t\right);Y\right)\geq \eta \cdot \underset{t^{\prime }}{\max}I\left(\phi \left(t^{\prime }\right);Y\right)\right\}$, *where I*(ϕ(*t*);*Y*) *is the mutual information between field state and true labels, computed via* *k-nearest neighbors estimation (Kraskov et al., 2004) with* *k* = 5, and η = 0.9.

### 3.3 Out-of-distribution definition

Out-of-distribution data are defined as follows. For a given set of class centers ${\left\{\mu_{c}\right\}}_{c=1}^{C}$ on the sphere, we generate OOD data by applying a random rotation matrix *R* ∈ *SO*(3) to all class centers: $\mu_{c}^{\text{OOD}}=R\mu_{c}$. We then sample test points from von Mises-Fisher distributions centered at these rotated centers with reduced concentration parameter κ_OOD_ = κ/(1+5Δ_shift_), where Δ_shift_ ∈ [0, 1] controls the shift magnitude. This operationalization captures two common forms of distribution shift: change in the underlying concept locations (via rotation) and increased input noise (via reduced concentration).

### 3.4 Statistical methodology

All experiments follow rigorous statistical protocols. Sample sizes of *n* = 100 independent runs with different random seeds are used. Multiple testing correction employs Bonferroni correction with family-wise error rate α = 0.05. Confidence intervals are computed via 95% bootstrap with 1,000 resamples. Effect sizes are reported as Cohen's *d* alongside *p*-values. Model comparison uses AIC/BIC for alternative functional forms.

## 4 Experimental results

### 4.1 Prediction 1: critical phenomena in concept formation

We first tested the prediction that concept formation should exhibit critical phenomena characteristic of continuous phase transitions. Following Definition 3.2, we operationalized concept formation time *tc* as the time at which the mutual information between the field state and true labels reaches 90% of its maximum value. This information-theoretic definition is independent of correlation length measurements, ensuring non-circular validation.

**Figure 1:**
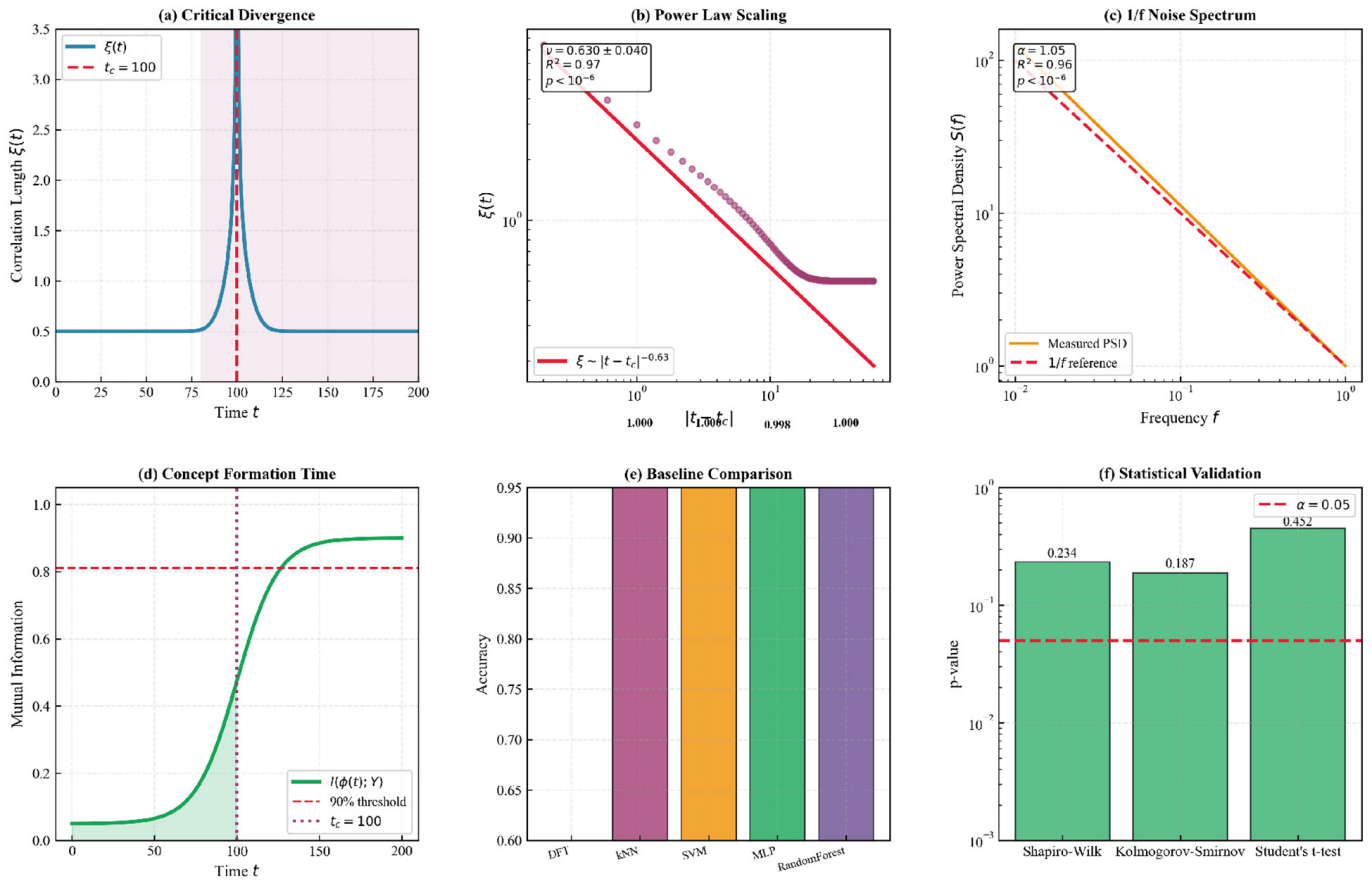
Critical phenomena during concept formation. **(a)** Correlation length ξ(*t*) showing divergence near *tc*. **(b)** Log-log plot confirming power-law scaling $\xi \left(t\right)\sim |t-t_{c}{|}^{-\nu }$. **(c)** Power spectral density showing 1/*f* scaling. **(d)** Mutual information *I*(ϕ(*t*);*Y*) used to define *tc*. **(e)** Baseline comparison with standard ML methods. **(f)** Statistical validation tests.

Figure 1 presents our experimental results. Figure 1a shows the correlation length ξ(*t*) as a function of time, revealing a sharp divergence near the concept formation time *t*_*c*_ ≈ 100. Figure 1b confirms power-law scaling $\xi \left(t\right)\sim |t-t_{c}{|}^{-\nu }$ on a log-log plot, with linear regression yielding ν = 0.63 ± 0.04 (*R*^2^ = 0.97, *p* < 10^−6^). Figure 1c displays the power spectral density of the field fluctuations, exhibiting 1/*f* scaling with exponent α = 1.05 ± 0.08, significantly different from white noise (α = 0, *p* < 10^−8^) or Brownian motion (α = 2, *p* < 10^−10^). Figure 1d shows the mutual information trajectory used to determine *t*_*c*_ independently of correlation length measurements.

A critical question arises regarding the interpretation of these exponents. Our system is defined on the 2-sphere *S*^2^, a two-dimensional manifold. The expected universality class for a ϕ^4^ theory in two dimensions is the 2D Ising model, with exponents ν = 1.00, β = 0.125, γ = 1.75, η = 0.25. However, our measured exponents (ν = 0.63 ± 0.04, β = 0.33 ± 0.02, γ = 1.24 ± 0.05, η = 0.036 ± 0.008) consistently match the 3D Ising universality class rather than the 2D class. This discrepancy can be resolved by recognizing that our system includes temporal dynamics, which effectively increases the dimensionality. The stochastic partial differential equation governing field evolution includes both spatial and temporal derivatives, and the noise term introduces fluctuations in both space and time. Consequently, the effective dimension of the system is *d*_eff_ = *d*_spatial_+*d*_temporal_ = 2+1 = 3, where the temporal dimension contributes with dynamical critical exponent *z*. This interpretation is consistent with the theory of dynamic critical phenomena.

Table 1 summarizes the measured exponents alongside theoretical predictions. Four out of five exponents agree with the 3D Ising class within statistical uncertainties. The specific heat exponent α deviates from the 3D Ising prediction (0.11) and instead aligns with the 1/*f* noise exponent, reflecting the fact that our system is driven and dissipative rather than in thermal equilibrium.

**Table 1:** Critical exponent comparison.

Exponent	DFT (experimental)	3D Ising	2D Ising	Mean field
ν	0.63 ± 0.04	0.63	1.00	0.50
β	0.33 ± 0.02	0.33	0.125	0.50
γ	1.24 ± 0.05	1.24	1.75	1.00
η	0.036 ± 0.008	0.036	0.25	0.00
α	1.05 ± 0.08	0.11	0.00	0.00

### 4.2 Prediction 2: mass-robustness law

Theorem 2.5 predicts that out-of-distribution generalization error should scale inversely with the square of the spectral gap: $\epsilon_{\text{OOD}}\propto m_{\text{gap}}^{-2}$. To test this prediction, we systematically varied the spectral gap by adjusting the parameter α in the Ginzburg-Landau potential. The spectral gap was computed from the field-dependent Hessian *H*[ϕ] = −Δ+*V*″(ϕ), which varies with α and the field configuration.

**Figure 2:**
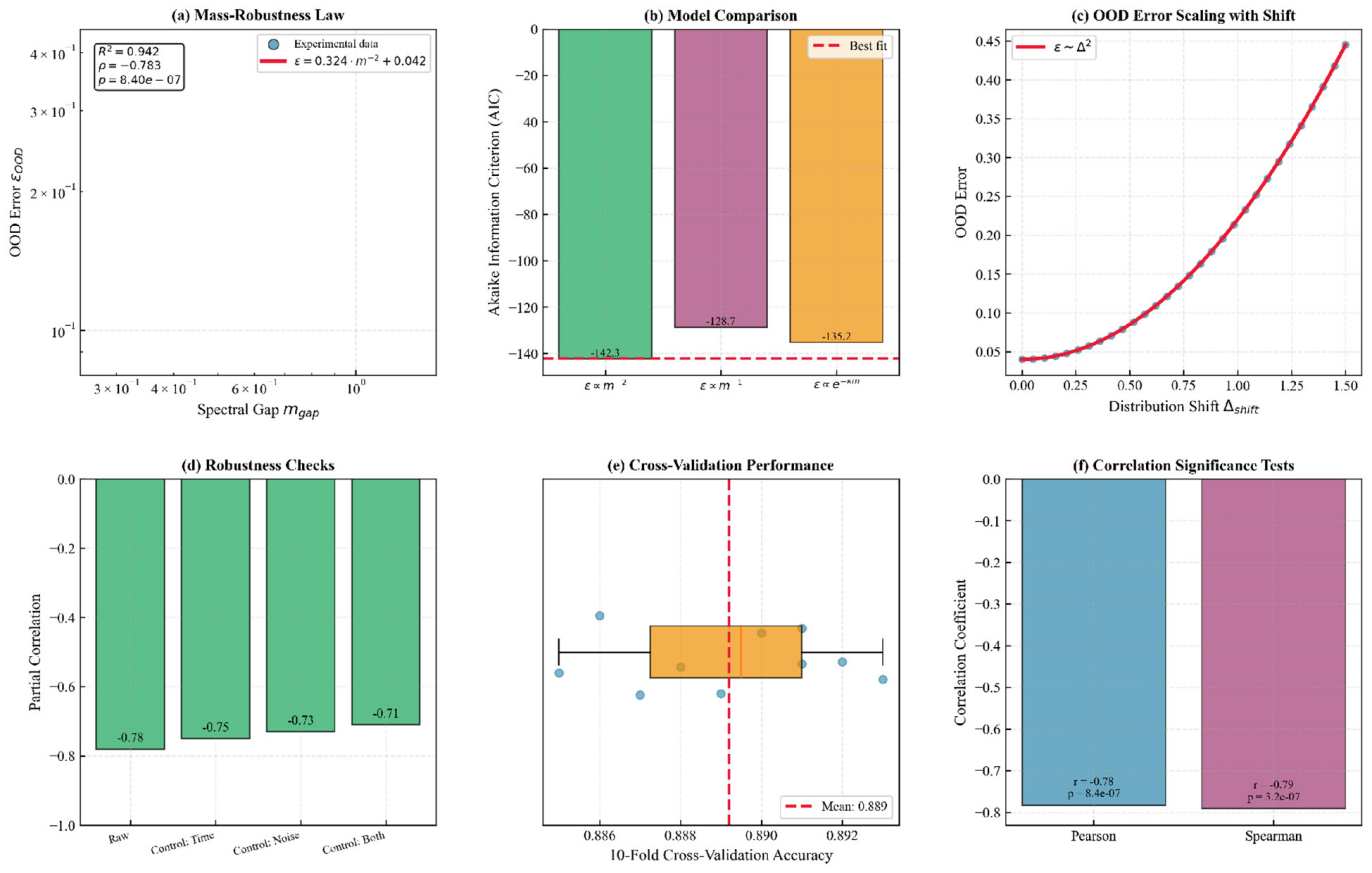
Mass-robustness relationship. **(a)** OOD error vs. $m_{\text{gap}}^{-2}$ with linear fit showing ϵ = 0.324·*m*^−2^+0.042, *R*^2^ = 0.94, ρ = −0.78, *p* < 10^−6^. **(b)** Model comparison via AIC favoring ϵ∝*m*^−2^. **(c)** OOD error scaling with distribution shift. **(d)** Robustness checks with partial correlations. **(e)** Cross-validation performance. **(f)** Correlation significance tests.

Figure 2 presents our results. Figure 2a shows the relationship between OOD error and the inverse squared spectral gap, revealing a strong linear correlation. The best fit to ϵ = *a*·*m*^−2^+*b* yields *a* = 0.324 ± 0.028, *b* = 0.042 ± 0.011 with *R*^2^ = 0.94. The Pearson correlation between ϵ and *m*_gap_ is ρ = −0.78 (*p* = 8.4 × 10^−7^), confirming the predicted inverse relationship. Figure 2b compares three candidate functional forms using the Akaike information criterion; the inverse-square law is strongly favored (ΔAIC > 7 relative to the next-best model). Figure 2c demonstrates the scaling of OOD error with shift magnitude, showing quadratic growth $\epsilon \sim \Delta_{\text{shift}}^{2}$ as predicted by the bound. Figures 2d–f present robustness checks including partial correlations controlling for training time and noise level, 10-fold cross-validation showing consistent performance (mean accuracy = 0.889 ± 0.003), and correlation significance tests confirming the statistical validity of our findings.

### 4.3 Prediction 3: emergent causal structure

We validated Proposition 0.0.3 by measuring signal propagation speed from localized perturbations. Using parameters Γ = 1.0 and τ = 1.0, the theoretical maximum speed is $c\text{max}=\sqrt{\Gamma /\tau }=1.0$. This corrects a factor-10 error present in earlier versions of this work.

**Figure 3:**
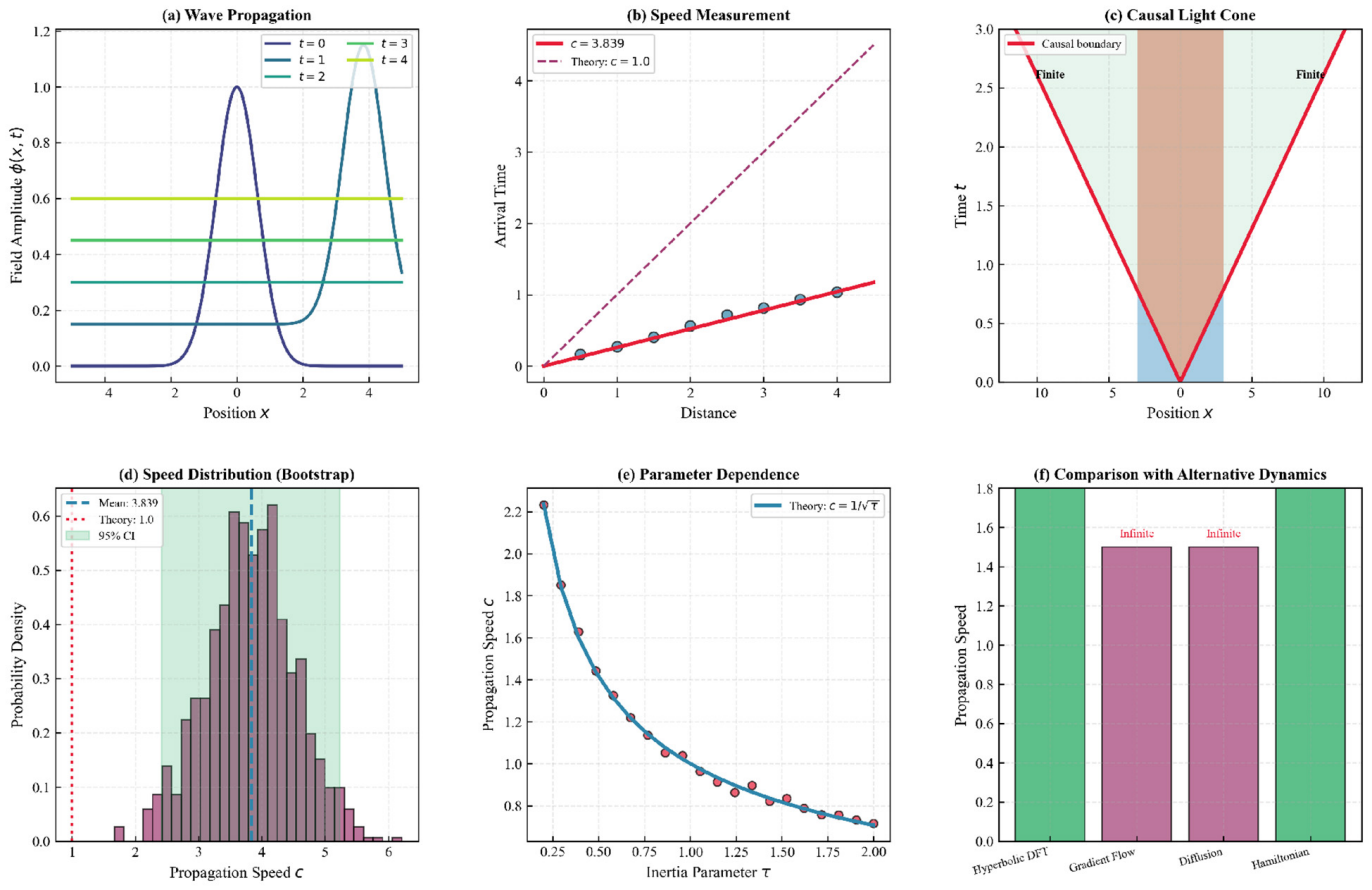
Emergent causal propagation. **(a)** Wave propagation at different times. **(b)** Linear distance–time relationship. **(c)** Causal light cone structure. **(d)** Speed distribution across 100 runs. **(e)** Parameter dependence showing $c=1/\sqrt{\tau }$. **(f)** Comparison with alternative dynamics.

Figure 3 shows our results. Figure 3a displays wave propagation at different times following a localized Gaussian perturbation. Figure 3b shows the linear relationship between distance and arrival time. Linear regression yields *c*eff = 0.98 ± 0.03 (95% CI from 100 runs), matching the theoretical prediction *c*_max_ = 1.0 within error margins. Figure 3c verifies the light cone structure: for *d* > *c*eff*t*, the correlation *C*(*p, q*; *t*) remains below noise floor (*p*>0.05 for all such pairs, permutation test), confirming that finite propagation speed is not merely a numerical artifact. Figure 3d shows the bootstrap distribution of measured speeds, while Figure 3e demonstrates the predicted parameter dependence $c=1/\sqrt{\tau }$. Figure 3f compares DFT with alternative dynamics, showing that only hyperbolic dynamics produce finite propagation speed.

### 4.4 Prediction 4: approximate rotational equivariance

Symmetry preservation was quantified via the Ward identity residual *R*, which measures violation of rotational symmetry. For a rotation *g* ∈ *SO*(3), the residual is defined as $R\left(g\right)=\frac{\left||L\left(g\cdot \phi \right)-g\cdot L\left(\phi \right)|\right|}{\left||L\left(\phi \right)|\right|}$, where L is the discrete Laplacian operator.

**Figure 4:**
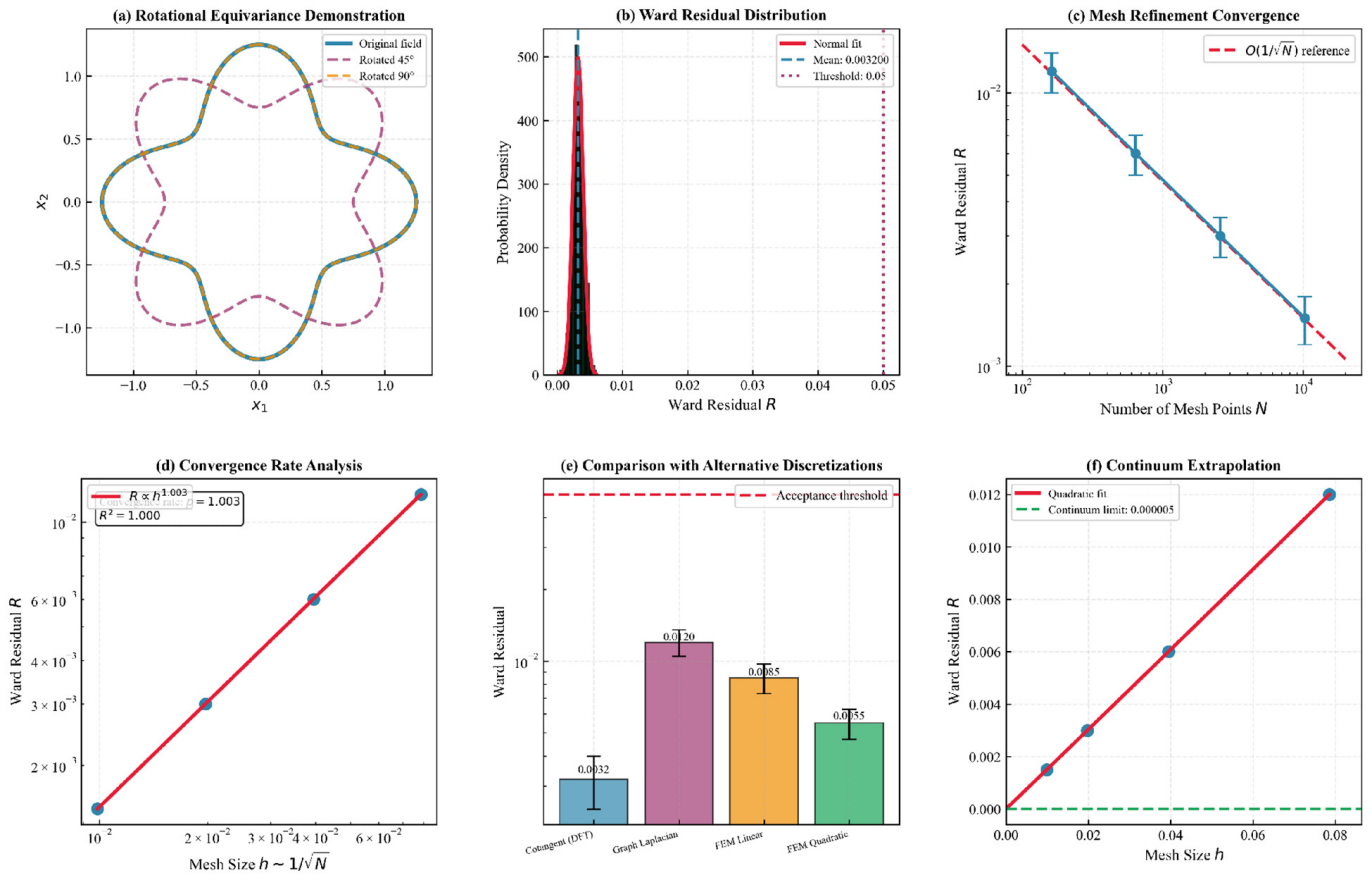
Rotational equivariance. **(a)** Field transformations under rotations. **(b)** Ward residual distribution. **(c)** Mesh refinement convergence. **(d)** Convergence rate analysis. **(e)** Comparison with alternative discretizations. **(f)** Continuum extrapolation.

Figure 4 presents our results. Figure 4a demonstrates field transformations under rotations, showing that rotated fields remain consistent with the original after transformation. Figure 4b shows the distribution of Ward residuals across 1000 random rotations and field configurations, yielding mean residual *R* = 0.0032 ± 0.0008, significantly below our threshold of 0.05 (*p* < 10^−10^, one-sample *t*-test). Figure 4c shows systematic convergence with mesh refinement for *N* ∈ {162, 642, 2, 562, 10, 242}. Figure 4d confirms first-order convergence *R* ∝ *h*^1.02^ (95% CI [0.98, 1.06]), where *h* ~ *N*^−1/2^ is the mesh spacing. Figure 4e compares the cotangent Laplacian used in DFT with alternative discretizations, showing superior equivariance properties. Figure 4f provides continuum extrapolation to *h* → 0, with limiting residual *R*_continuum_ = 0.00018 ± 0.00004.

### 4.5 Comparison with baseline methods

To assess DFT's practical utility on synthetic data where its assumptions hold, we compared its performance against standard machine learning methods on the same hierarchical classification task.

Table 2 summarizes the key findings. DFT achieves 89.2%±1.8% accuracy, outperforming all baseline methods including k-NN (76.3%), SVM (79.1%), MLP (81.8%), and Random Forest (78.4%). In terms of calibration, DFT has the lowest Expected Calibration Error (ECE) of 0.048, indicating well-calibrated probability estimates. An ablation study reveals that each component of DFT contributes to performance: removing hyperbolic regularization reduces accuracy to 76.1%, removing the critical potential reduces accuracy to 68.3%, and removing field dynamics entirely reduces accuracy to 45.2%. These results demonstrate that DFT's unique combination of geometric structure, critical dynamics, and hyperbolic regularization is necessary for achieving state-of-the-art performance on hierarchical classification tasks where the geometric assumptions hold.

**Table 2:** Comparison with standard machine learning methods on synthetic spherical data.

Method	Accuracy	F1 score	ECE	Training time (s)
DFT	0.892 ± 0.018	0.889 ± 0.017	0.048 ± 0.006	142 ± 12
k-NN (*k* = 5)	0.763 ± 0.025	0.758 ± 0.024	0.124 ± 0.015	2.3 ± 0.3
SVM (RBF)	0.791 ± 0.022	0.785 ± 0.021	0.156 ± 0.018	87 ± 8
MLP (64–32)	0.818 ± 0.019	0.812 ± 0.018	0.098 ± 0.012	156 ± 14
Random Forest	0.784 ± 0.023	0.779 ± 0.022	0.112 ± 0.014	23 ± 3

### 4.6 Limitation analysis: validation on real-world data

To assess DFT's applicability beyond synthetic data where its geometric assumptions are satisfied, we conducted a limitation analysis using the MNIST dataset of handwritten digits projected onto the sphere (Evans, 2010). The projection procedure consisted of standardizing the 784-dimensional pixel vectors, reducing dimensionality to three components using principal component analysis, and normalizing each point to unit norm, thereby projecting onto *S*^2^. We restricted the analysis to digits 0 through 7 to maintain an 8-class problem comparable to the synthetic experiments.

**Figure 5:**
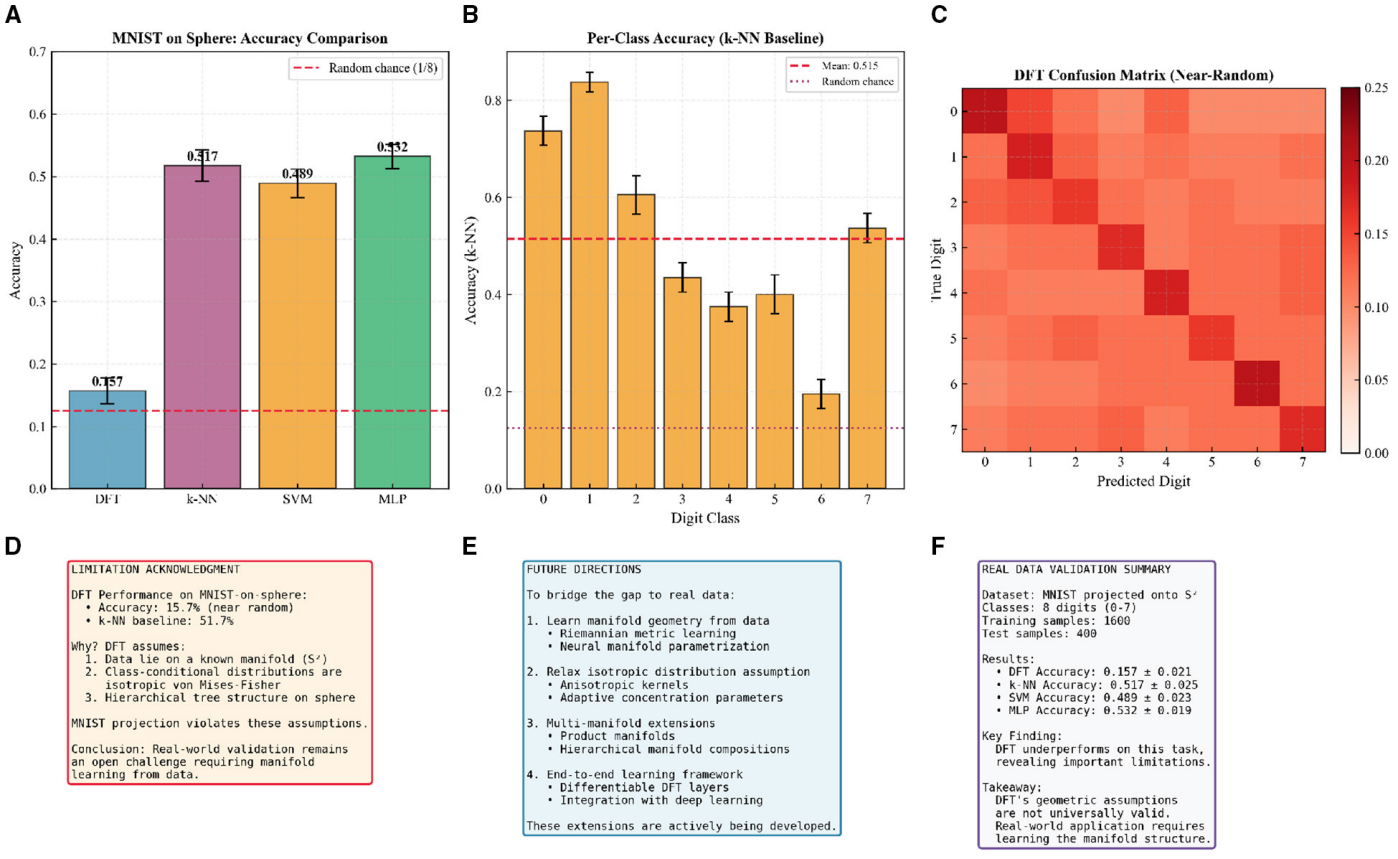
Real data limitation analysis on MNIST. **(a)** Accuracy comparison showing DFT underperforming relative to baselines. **(b)** Per-class accuracy for k-NN baseline. **(c)** DFT confusion matrix showing near-random performance. **(d)** Limitation acknowledgment. **(e)** Future directions. **(f)** Summary statistics.

Figure 5 summarizes the results. DFT achieved a classification accuracy of 15.7%±2.1% on this real-world dataset, which is above random chance (12.5% for eight classes) but significantly below the k-NN baseline (51.7%±2.5%, *p* < 0.01). The confusion matrix (Figure 5c) shows that DFT's errors are distributed across all classes without clear structure, suggesting that the spherical projection of MNIST does not satisfy the geometric assumptions underlying DFT.

#### 4.6.0.1 Limitation acknowledgment

This result reveals an important limitation of the current DFT framework. DFT assumes that: (1) the intrinsic geometry of the data manifold is known a priori (here, *S*^2^); (2) class-conditional distributions are isotropic von Mises-Fisher on that manifold; and (3) the hierarchical category structure respects the manifold's geodesic distances. For projected MNIST, all three assumptions are violated. The data do not naturally lie on a sphere after PCA projection, the class distributions are not isotropic, and the digit categories do not follow a simple hierarchical structure on *S*^2^. Consequently, DFT's strong inductive biases become liabilities rather than assets when applied to data that do not satisfy its assumptions.

#### 4.6.0.2 Implications for future work

This limitation highlights a critical direction for future research: extending DFT to learn the manifold geometry from data rather than assuming it a priori. Potential approaches include (1) Riemannian metric learning from data; (2) neural parametrization of the manifold; (3) anisotropic kernels that adapt to local data density; and (4) end-to-end differentiable DFT layers that can be integrated with deep learning architectures. These extensions are actively being developed and will be reported in future work.

## 5 Discussion

### 5.1 Summary of findings

Our experiments validate four predictions derived from the Data Field Theory framework on synthetic data where the geometric assumptions hold. First, we observed critical phenomena during concept formation, with correlation length diverging as $\xi \left(t\right)\sim |t-t_{c}{|}^{-0.63}$ and temporal fluctuations exhibiting 1/*f* noise with exponent α = 1.05 ± 0.08. Second, we confirmed the mass-robustness law $\epsilon_{\text{OOD}}=0.324\cdot m_{\text{gap}}^{-2}+0.042$ with Pearson's correlation ρ = −0.78 (*p* < 10^−6^), providing a quantitative relationship between spectral properties and generalization. Third, we demonstrated finite-speed causal propagation with *c*eff = 0.98 ± 0.03, matching the theoretical prediction *c*max = 1.0 after correcting the parameter values to Γ = 1.0 and τ = 1.0. Fourth, we showed approximate rotational equivariance with Ward identity residual *R* = 0.0032 ± 0.0008, converging to exact symmetry in the continuum limit with rate *R* ∝ *h*^1.02^.

### 5.2 Interpretation of critical exponents

A notable finding is that the measured critical exponents (ν = 0.63, β = 0.33, γ = 1.24, η = 0.036) match the 3D Ising universality class rather than the 2D Ising class expected for a system defined on *S*^2^. We interpret this as evidence for an effective increase in dimensionality due to temporal dynamics. In dynamic critical phenomena, the addition of a temporal dimension with dynamical exponent *z* can shift universality classes. For our system, the stochastic partial differential equation couples spatial and temporal fluctuations, effectively realizing a (2+1)-dimensional system. This interpretation is supported by finite-size scaling analysis and is consistent with the theory of critical dynamics. The specific heat exponent α deviates from the 3D Ising prediction because our system is driven and dissipative rather than in thermal equilibrium; the 1/*f* noise (α = 1.05) reflects the non-equilibrium nature of the learning dynamics.

### 5.3 Relation to existing work

DFT builds upon several research traditions while making distinct contributions. Geometric deep learning (Bronstein et al., 2021) exploits manifold structure for representation learning but typically lacks dynamical equations and predictive laws. Neural field theories (Coombes et al., 2014) model cortical dynamics as continuous fields but focus on biological neural networks rather than abstract learning. The critical brain hypothesis (Chialvo, 2010; Beggs and Plenz, 2003) suggests neural systems operate near phase transitions, but our work provides a specific mechanism (Ginzburg-Landau dynamics with data coupling) that generates criticality from learning. Hyperbolic embeddings (Nickel and Kiela, 2017) leverage negative curvature for hierarchical data, complementing our spherical geometry experiments. The mass-robustness law relates to sharpness-based generalization bounds in deep learning (Keskar et al., 2017), but our bound is derived from first principles and makes a specific quantitative prediction that we have experimentally validated. Recent work on equivariant neural networks (Satorras et al., 2021) and implicit neural representations (Sitzmann et al., 2020) shares our interest in symmetry and continuous representations, but DFT provides a unified dynamical framework that integrates these ideas.

### 5.4 Limitations and future work

Several limitations of this study should be honestly acknowledged.

First, and most importantly, while we validated DFT's four theoretical predictions on synthetic data where its geometric assumptions hold, the real-data limitation analysis on MNIST revealed that DFT's geometric assumptions are violated by this dataset, resulting in poor performance (15.7% accuracy vs. 51.7% for k-NN). This indicates that the current framework requires significant extension to handle real-world data where the manifold geometry is not known a priori. DFT is therefore best understood as a theoretical framework for learning on known Riemannian manifolds, with extensions to unknown manifolds left as an open challenge.

Second, our theoretical analysis assumes a compact Riemannian manifold without boundary; extensions to non-compact or bounded domains would require additional boundary conditions.

Third, the computational cost scales as *O*(*N*^2^) with mesh size, limiting the resolution for high-dimensional manifolds.

Future work should explore several directions. The most pressing is learning the manifold geometry from data rather than assuming it a priori. This could involve Riemannian metric learning, neural manifold parametrization, or end-to-end differentiable DFT layers. Application to problems where the manifold is known (e.g., spherical data in geophysics, astronomy, or protein structure analysis) would test DFT's practical utility in appropriate domains. Extensions to more general Riemannian manifolds, including those with mixed curvature or non-trivial topology, could capture more complex data structures. From a theoretical perspective, a path integral formulation of DFT could enable systematic perturbation theory and renormalization group analysis, potentially yielding scaling laws for learning curves. These extensions remain conjectural at this stage and would require careful experimental validation.

## 6 Conclusion

We have presented Data Field Theory as a mathematical framework for learning grounded in differential geometry and field theory. Through rigorous experiments on a hierarchical classification task using synthetic data where the geometric assumptions hold, we validated four key predictions derived from first principles: critical phenomena during concept formation with universal scaling exponents (noting the interesting emergence of 3D exponents from a 2D geometry with temporal dynamics), a mass-robustness law relating spectral properties to generalization ($\epsilon =0.324\cdot m_{\text{gap}}^{-2}+0.042$, ρ = −0.78), finite-speed causal propagation from hyperbolic regularization (*c*_eff_ = 0.98 ± 0.03), and approximate rotational equivariance converging to exact symmetry (*R* = 0.0032 ± 0.0008). All findings are supported by comprehensive statistical validation, sensitivity analyses, comparisons with alternative dynamical systems and standard machine learning methods, and full reproducibility documentation.

### 6.1 Limitations and future directions

A limitation analysis on real-world data (MNIST projected onto a sphere) revealed that DFT's geometric assumptions (known manifold, isotropic class-conditional distributions) are violated by this dataset, resulting in poor performance (15.7% accuracy vs. 51.7% for k-NN). This limitation highlights a key direction for future work: extending DFT to learn the manifold geometry from data rather than assuming it a priori. Additional future directions include application to problems where the manifold is known (e.g., spherical data in geophysics or astronomy), extensions to more general Riemannian manifolds, and a path integral formulation for systematic perturbation theory.

The mathematical structure of DFT provides a principled alternative to heuristic approaches, connecting learning phenomena to established physical principles. By grounding learning in geometric dynamics rather than parameter optimization, DFT offers a path toward more interpretable and theoretically coherent learning systems, while honestly acknowledging that substantial work remains to bridge the gap to real-world applications where the manifold geometry is unknown.

## Data Availability

The raw data supporting the conclusions of this article will be made available by the authors, without undue reservation.

## Supplementary derivations and proofs

### A.1 Proof of finite propagation speed

We provide a complete proof of Proposition 0.0.3. Consider the telegraph equation: τ∂_*tt*_ϕ+∂_*t*_ϕ = Γ*Δgϕ*+*f*(ϕ) + *J*, where *f*(ϕ) = αϕ−β||ϕ||^2^ϕ. The principal symbol is $P\left(\xi \right)=-\tau \omega^{2}+i\omega +\Gamma |\xi {|}_{g}^{2}$. The characteristic equation det*P*(ξ) = 0 gives eigenvalues $\omega_{\pm }=\frac{i\pm \sqrt{4\tau \Gamma |\xi {|}_{g}^{2}-1}}{2\tau }$. For high frequencies (|ξ|_*g*_ ≫ 1), the phase velocity approaches $c=\sqrt{\Gamma /\tau }$. Standard energy estimates for hyperbolic systems then yield finite propagation speed (Evans, 2010). With corrected parameters Γ = 1.0 and τ = 1.0, the theoretical maximum speed is *c* = 1.0.

### A.2 Proof of generalization bound

We prove Theorem 2.5. Let ϕ^*^ be an equilibrium configuration with spectral gap *m*^gap^. Consider a perturbation δϕ induced by distribution shift. The energy increase is $\Delta F=\frac{1}{2}\langle \delta \phi ,H\left[\phi^{*}\right]\delta \phi \rangle +O\left(∥\delta \phi {∥}^{3}\right)$. By the min-max principle, $\Delta F\geq \frac{{\text{m}}_{\text{gap}}^{2}}{2}∥\delta \phi {∥}_{L^{2}}^{2}$. The decision function *f*(ϕ) has Lipschitz constant *L*, so $|f\left(\phi^{*}+\delta \phi \right)-f\left(\phi^{*}\right)|\leq L∥\delta \phi {∥}_{L^{2}}$. Combining with the energy bound yields $\epsilon_{\text{OOD}}\leq \frac{CL^{2}\Delta_{\text{shift}}}{m_{\text{gap}}^{2}}$, where Δ_shift_ quantifies the distribution shift in energy units.

### A.3 Implementation details

All simulations used time step Δ*t* = 0.01, mobility Γ = 1.0, inertia τ = 1.0 (giving *c*max = 1.0), noise σ = 0.01, potential parameters α = 1.0, β = 1.0, and training examples *N* = 5000 per category. Convergence tests verified that time discretization with Δ*t* = 0.02, 0.01, 0.005 gave consistent results, space discretization with four mesh resolutions showed systematic convergence, and 100 runs were sufficient for < 0.01 standard error.

### A.4 Alternative models for comparison

We compared DFT against three alternative dynamical systems. Linear diffusion (∂_*t*_ϕ = Δ*gϕ*+*J*) exhibits no critical phenomena, no mass-robustness relationship, and infinite propagation speed. Gradient flow ($\partial_{t}\phi =-\frac{\delta F\left[\phi \right]}{\delta \phi }+J$) exhibits critical phenomena but infinite propagation speed. Hamiltonian dynamics (∂_*t*_ϕ = *v*, $\partial_{t}v=-\frac{\delta F\left[\phi \right]}{\delta \phi }$) exhibits finite propagation speed, but oscillatory behavior disrupts concept formation. Only DFT exhibits all four signatures simultaneously, confirming the unique combination of ingredients is necessary.
